# Determining the Cancer Center priorities at the Wilmot Cancer Institute: A proposed framework informed by an academic-community collaborative approach

**DOI:** 10.1093/oncolo/oyaf282

**Published:** 2025-09-15

**Authors:** Francisco Cartujano-Barrera, Ana Paula Cupertino, Emily Hayes, Candice Lucas, Electra D Paskett, Erin Kobetz, Jeffrey Freeman, Karen Hermance, Nikisha Ridgeway, Charles S Kamen

**Affiliations:** Wilmot Cancer Institute, University of Rochester Medical Center, Rochester, NY, United States; Department of Public Health Sciences, University of Rochester Medical Center, Rochester, NY, United States; Wilmot Cancer Institute, University of Rochester Medical Center, Rochester, NY, United States; Department of Surgery, University of Rochester Medical Center, Rochester, NY, United States; Wilmot Cancer Institute, University of Rochester Medical Center, Rochester, NY, United States; Equity and Advocacy Division, Urban League of Rochester, Rochester, NY, United States; Department of Internal Medicine, The Ohio State University, Columbus, OH, United States; Department of Medicine, University of Miami School of Medicine, Miami, FL, United States; Common Ground Health, Rochester, NY, United States; The Sally Edelman and Harry Gardner Cancer Research Foundation, Rochester, NY, United States; Starbridge, Rochester, NY, United States; Wilmot Cancer Institute, University of Rochester Medical Center, Rochester, NY, United States; Department of Surgery, University of Rochester Medical Center, Rochester, NY, United States

**Keywords:** Cancer Center, national cancer institute, Cancer Center priorities, academic-community collaborative approach

## Abstract

Current National Cancer Institute (NCI) Cancer Center Support Grant guidelines include a Community Outreach and Engagement merit descriptor related to the justification of Cancer Center priorities. Unfortunately, there is limited guidance and published literature on the process of determining the Cancer Center priorities. The purpose of this commentary is to propose a framework for Cancer Center prioritization, informed by the academic-community collaborative approach that the Wilmot Cancer Institute (Wilmot) at the University of Rochester implemented as part of a successful application for NCI designation. We first defined Wilmot’s catchment area and curated data on the cancer burden in that area. We then collaborated with program leaders to assess Wilmot’s capacity to address the cancer burden in its catchment area. We iteratively worked with our Community Cancer Action Council (CCAC) to determine the Cancer Center priorities. Cancer incidence, mortality, risk factors, and screening data, as well as ongoing research from Wilmot’s scientific programs, were the factors that Wilmot leaders and CCAC partners considered to make this determination. Thus, (1) tobacco-related cancers (ie, bladder, esophageal, head and neck, larynx, and lung cancers; including addressing tobacco prevention and cessation, and promoting lung cancer screening), (2) hematologic malignancies (ie, leukemia and lymphoma), and (3) pancreatic and hepatobiliary cancers were determined as the Cancer Center priorities. These Cancer Center priorities have informed research and outreach at Wilmot.

## Introduction

Community Outreach and Engagement (COE) is a required component of the National Cancer Institute (NCI) Cancer Center Support Grant (CCSG).^1^^,^^2^ Under this component, Cancer Centers are expected to ensure the research they conduct is relevant and responsive to the needs of the communities they serve. The assessment of COE is strongly correlated with Cancer Centers’ overall CCSG review score.^3^ Current CCSG guidelines include a COE merit descriptor related to the justification of Cancer Center priorities: “How well has the Center identified and prioritized the cancer research and control needs of its catchment area population?”^1^ Unfortunately, there is limited guidance and published literature on the process of determining the Cancer Center priorities. The purpose of this commentary is to propose a framework for Cancer Center prioritization, informed by the academic-community collaborative approach that the Wilmot Cancer Institute (Wilmot) implemented as part of a successful application for NCI designation.

## Defining the catchment area

**Figure 1.**
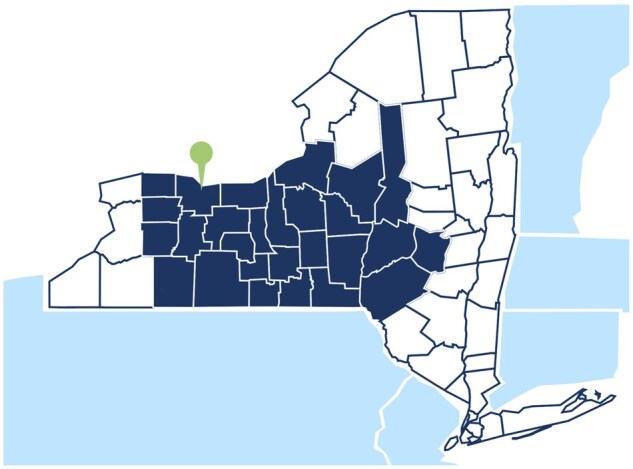
The Wilmot catchment area.

The NCI mandates that Cancer Centers define their catchment area (CA), or the population and geographic region they serve, to identify factors that influence the cancer burden in that area and deploy cancer prevention and control activities.^1^^,^^2^^,^^4–6^ ­Wilmot defined its CA by mapping new patients seen for care as well as those accrued to clinical trials. In 2022, Wilmot provided care and clinical trial options to patients from 27 counties in Western and Central NY State (NYS), and 97% of the patients seen at Wilmot were drawn from the CA. The CA population comprises 3,154,521 residents (Figure 1).

## Examining data on cancer incidence, mortality, risk factors, and screening

The overall age-adjusted cancer incidence rate in the Wilmot CA is strikingly high, at 510.9 cases per 100,000 residents. This is higher than NYS and US rates (479.5 cases and 445.5 cases per 100,000, respectively). If the Wilmot CA were a US state, it would have the second-highest cancer incidence rate in the nation, following Kentucky. Rates of 9 out of 10 common cancers are higher in the Wilmot CA than in NYS and the USA (Table S1).

Similarly, the Wilmot CA has a higher age-adjusted cancer mortality rate (158.3 deaths per 100,000 residents) than NYS and USA rates (141.0 deaths and 152.4 deaths per 100,000, respectively). Mortality rates for tobacco-related (e.g., lung, esophageal), hematologic (e.g., lymphoma, leukemia), and pancreatic cancers are higher in the CA than in NYS and the USA (Table S2), and racial/ethnic disparities are notable.

The Wilmot CA is burdened by important cancer risk factors. For example, rates of cigarette smoking are higher in the Wilmot CA than in the rest of the nation (Table S3). Smoking rates among Black (25%) and Latino (19%) adults are disproportionately high. Rates of HPV vaccination among those aged 13-17 in the CA are significantly below NYS levels, particularly in rural counties.

NYS goals are for 75% of individuals aged 40-74 who are eligible for breast cancer screening to receive a mammogram, 86% of individuals aged 21-65 with a cervix to receive a cervical cancer screening, and 80% of people aged 50-75 to receive colorectal cancer screening. The majority of counties in the CA are not meeting the goals for breast, cervical, and colorectal cancer screening. Rates of early-stage detection of lung cancer in the CA are also below NYS levels (Table S3).

## Engaging diverse communities through the Community Cancer Action Council

To engage diverse communities across the CA, Wilmot partnered with a Community Cancer Action Council (CCAC). The CCAC serves as Wilmot’s community advisory board. The CCAC includes community partners representing more than 20 community organizations from across the CA. The missions and services of these community organizations reflect the demographic composition and needs of the CA.

CCAC members meet monthly in virtual, open, town-hall-style meetings in support of a shared mission, “to work with communities today to lessen the impact of cancer tomorrow.” The CCAC was engaged early in the process of defining the CA and has been included in an ongoing fashion as COE leaders curated and refined data on the cancer burden.

## Reviewing Wilmot’s scientific programs

COE leaders collaborated with program leaders to assess Wilmot’s capacity to address the cancer burden in its CA. This collaboration informed a comprehensive analysis of Wilmot’s research portfolio. Investigators at Wilmot are organized into three interactive scientific programs: (1) Genetics, Epigenetics and Metabolism, (2) Cancer Microenvironment, and (3) Cancer Prevention and Control (CPC). Three major themes emerged across the three scientific programs: (1) tobacco-related cancers, (2) hematologic malignancies, and (3) pancreatic and hepatobiliary cancers. In 2021, of all Wilmot research funding, 35% (more than $9 million direct costs annually) related to one of these themes. Wilmot investigators are recognized internationally for their scientific accomplishments within these cancer areas.

## Determining the Cancer Center priorities

In 2020 (for the first submission of the CCSG application) and again in 2023 (for the resubmission), COE leaders aggregated and presented data on cancer incidence, mortality, risk factors, and screening to CCAC partners. COE leaders also presented examples of ongoing research from Wilmot’s scientific programs. The data and research examples were conveyed through PowerPoint presentations featuring comprehensive visual elements. These materials were shared in real time via Zoom^®^ meetings and asynchronously via email. Ample time was allocated for questions during the live sessions, and email remained open for ongoing communication. COE leaders then facilitated discussions among CCAC partners to determine the Cancer Center priorities. The goal of these discussions was to foster clarification and mutual insight around shared priorities.

Through a consensus-based approach, CCAC partners collectively recommended that Wilmot continue to focus activities on cancers of high incidence (e.g., breast, prostate, colorectal) and elevated risk factors in the Wilmot CA (e.g., obesity). However, CCAC partners collectively recommended that Wilmot maintain specific effort to three priority cancer groups where the cancer burden is high and where Wilmot scientists are poised to make a strong impact. Thus, the Cancer Center priorities were determined as: (1) tobacco-related cancers (ie, bladder, esophageal, head and neck, larynx, and lung cancers; including tobacco prevention and cessation, and lung cancer screening), (2) hematologic malignancies (ie, leukemia and lymphoma), and (3) pancreatic and hepatobiliary cancers.

CCAC partners also emphasized the importance of focusing on specific populations in the CA that experience disparities: Black, Deaf, Latino, older adult, rural, and sexual and gender minority communities. This focus was recommended not as a separate Cancer Center priority, but as a critical cross-cutting theme that should shape and inform all research and outreach efforts.

## Informing research and outreach activities

The determination of Wilmot’s priorities has directly informed research and outreach activities. For instance, Wilmot has made significant investment in addressing tobacco-related cancers in the CA. In terms of research, Wilmot has recruited and supported investigators focused on expanding the tobacco control scientific portfolio. One example is Dr Cartujano-Barrera, an early career investigator from the CPC program. With Wilmot support, Dr Cartujano-Barrera, colleagues, and community members developed *Actívatexto*, a mobile intervention that promotes smoking cessation and physical activity among Latinos.^7–9^ A community-based pilot study demonstrated that *Actívatexto* increased levels of physical activity and resulted in a noteworthy cessation rate.^10^ These results informed a newly funded R01 to assess the efficacy of *Actívatexto*.

On the outreach front, in December 2020, Wilmot launched the Wilmot Tobacco Cessation Program, offering counseling via text messages and pharmacotherapies at no cost to community members across the CA. Since its launch, and in partnership with local clinics and community-based organizations, over 700 diverse patients have enrolled in the program. The program has achieved a cessation rate of over 40% at Month 3.

## Achieving NCI designation

In January 2024, Wilmot resubmitted a CCSG application to become a newly designated center, and in May 2024, Wilmot underwent a site visit. CCSG reviewers positively noted that “The determination of research and outreach priorities were guided by (cancer incidence, mortality, risk factors, and screening) data and ongoing research strengths from Wilmot’s scientific programs. This information was presented by COE leaders to CCAC partners who agreed with a prioritization of cancers with high burden that matched existing scientific strengths at Wilmot”. In March 2025, the NCI named Wilmot the nation’s 73^rd^ Designated Cancer Center.^11^

## Discussion

Dr Molina and colleagues described the academic-community collaborative approach that the University of Illinois implemented to determine its Cancer Center priorities.^12^ Both the University of Illinois and Wilmot established their Cancer Center priorities based on cancer incidence, mortality, risk factors, and screening data, along with extensive community input. However, as recommended by CCAC partners, ongoing research from the scientific programs was another key factor in determining the Cancer Center priorities at Wilmot.

The University of Iowa Health implemented an academic-community collaborative process to develop criteria for determining Cancer Center priorities.^13^ Their framework includes three core considerations: (1) whether patients face significant barriers to cancer treatment, screening, or clinical trial participation, (2) whether the priority involves a preventable cancer or is related to one, and (3) whether the priority addresses a health inequity. This focus on access, prevention, and equity represents a critical and commendable approach in ensuring responsiveness to community needs. Nonetheless, the absence of a criterion explicitly tied to ongoing scientific research represents a potential limitation.

### Framework for Cancer Center prioritization

**Figure 2.**
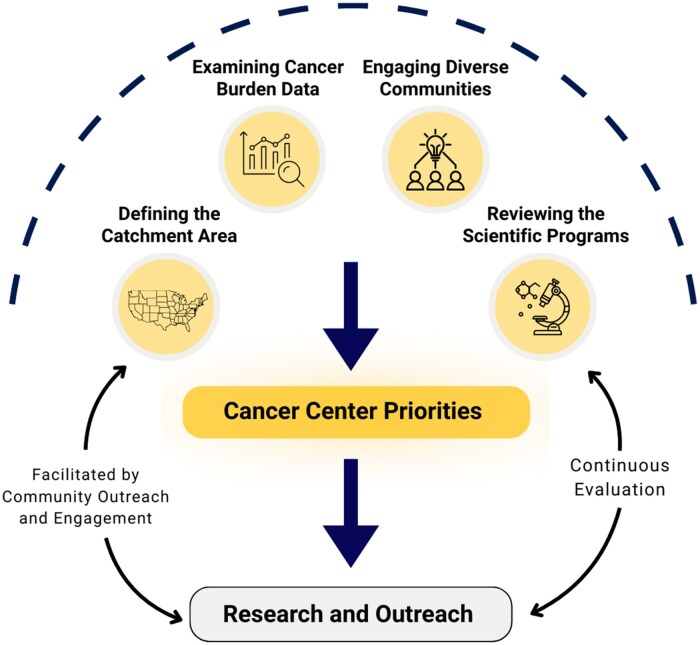
Framework for Cancer Center prioritization.

The proposed framework outlines four activities: (1) defining the CA, (2) examining data on cancer incidence, mortality, risk factors, and screening, (3) engaging diverse communities, and (4) reviewing the scientific programs (Figure 2). Then, through an iterative, consensus-based process facilitated by COE leaders, priorities should be established with community partners, integrating data on the cancer burden in the CA with the Cancer Center’s scientific capacity to address it. Lastly, the Cancer Center priorities should inform research and outreach activities. The proposed framework has the potential to ensure that Cancer Centers are poised to make a strong impact in their CA. The determination of Cancer Center priorities should be an ongoing process that requires continuous evaluation and community input.

## Conclusion

The proposed framework for Cancer Center prioritization was informed by the academic-community collaborative approach that Wilmot implemented as part of a successful application for NCI designation.

Cancer incidence, mortality, risk factors, and screening data, as well as ongoing research from Wilmot’s scientific programs were the factors that Wilmot leaders and CCAC partners considered to make this determination. Tobacco-related cancers, hematologic malignancies, and pancreatic and hepatobiliary cancers were determined the areas that Wilmot was poised to make a strong impact. These Cancer Center priorities have informed research and outreach at Wilmot.

## Supplementary Material

oyaf282_Supplementary_Data

## Data Availability

No data are associated with this article.
