# Supplementary material for: Determining the Cancer Center priorities at the Wilmot Cancer Institute: A proposed framework informed by an academic-community collaborative approach
Source: Oncologist. 2025 Sep 15;30(10):oyaf282. doi: 10.1093/oncolo/oyaf282 (PMC12517334; doi:10.1093/oncolo/oyaf282)
Supplement: oyaf282_Supplementary_Data [file oyaf282_supplementary_data.zip › Supplemental Tables.docx]

**Supplemental Table 1. Selected common cancer incidence rates in the Wilmot catchment area.**

|  | USA | NYS | CA Overall | CA Urban | CA Rural | CA White | CA Black | CA Overall |
| --- | --- | --- | --- | --- | --- | --- | --- | --- |
| **Breast** | 68.6 | 73.2 | **74.2** | **75.9** | 67.5 | **75.3** | 71.8 | 67.7 |
| **Lung** | 52.0 | 55.1 | **69.9** | 69.4 | 72.0 | 70.5 | **77.5** | 55.5 |
| **Prostate** | 51.7 | 58.3 | **63.0** | **64.7** | 56.5 | 61.6 | **96.9** | 58.6 |
| Colorectal | 37.7 | 37.6 | 38.2 | 37.2 | **42.1** | 38 | **44.7** | 34.8 |
| **Bladder** | 18.7 | 21.5 | **25.5** | 25.5 | 25.4 | 26.4 | 14.3 | 21.3 |
| **Melanoma** | 21.5 | 16.9 | **24.7** | 24.2 | **27.1** | 27.1 | 1.6 | 7.4 |
| **NH Lymphoma** | 19.0 | 21.6 | **20.9** | 21.3 | 19.7 | **21.3** | 16.4 | **24.1** |
| **Kidney** | 17.3 | 16.7 | **19.3** | 19.6 | 18.2 | 19.2 | **27.1** | 14.5 |
| **Leukemia** | 14.1 | 15.9 | **16.9** | 16.8 | 17.3 | 17.4 | 11.9 | 15.3 |
| **Pancreas** | 13.3 | 14.4 | **14.6** | 14.7 | 14.1 | 14.4 | **20.7** | 15.2 |
| **Note**: Shading indicates overall catchment area rates are significantly higher than either USA or NYS; Urban/Rural rates differ from one another; or rates by racial/ethnic group differ from one another. **Source**: SEER Registry | | | | | | | | |

**Supplemental Table 2. Selected common cancer mortality rates in the Wilmot catchment area.**

|  | USA | NYS | CA Overall | CA Urban | CA Rural | CA White | CA Black |
| --- | --- | --- | --- | --- | --- | --- | --- |
| **Lung** | 36.7 | 32.0 | **41.7** | 41.2 | 43.7 | 42.2 | 46.1 |
| **Colorectal** | 13.4 | 12.2 | 12.7 | 12.3 | 13.9 | 12.6 | **16.3** |
| **Pancreas** | 11.1 | 11.0 | **11.7** | 11.8 | 11.2 | 11.7 | **16.7** |
| **Breast** | 11.0 | 10.6 | 9.9 | 10.1 | 9.3 | 9.9 | 11.7 |
| **Prostate** | 7.8 | 6.9 | 7.2 | 7.0 | 7.9 | 7.0 | **12.0** |
| **Leukemia** | 6.1 | 5.9 | **6.6** | 6.6 | 6.6 | 6.6 | 6.4 |
| **Lymphoma** | 5.5 | 5.3 | **6.2** | 6.0 | 5.9 | 6.1 | 4.9 |
| **Liver** | 6.6 | 5.8 | 5.3 | 5.7 | 5.5 | 5.8 | 4.5 |
| **Esophagus** | 3.9 | 3.4 | **4.8** | 5.4 | 4.8 | 4.9 | 4 |
| **Brain** | 4.4 | 3.9 | 4.7 | 4.8 | 4.3 | **5.0** | 2.4 |
| **Note**: Shading indicates overall catchment area (CA) rates are significantly higher than either USA or NYS; Urban/Rural rates differ from one another; or rates by racial/ethnic group differ from one another. **Source**: SEER Registry | | | | | | | |

**Supplemental Table 3. Selected common cancer risk factors and screening rates in the Wilmot catchment area.**

|  | USA | NYS | CA |
| --- | --- | --- | --- |
| Alcohol drinking | 15.5% | 14.6% | 15.2% |
| **Cigarette smoking** | 14.2% | 11.1% | **16.9%** |
| Vaping | -- | 19.7% | 19.3% |
| **Obesity** | 31.9% | 25.2% | **32.6%** |
| Physical inactivity | 23.8% | 25.8% | 24.3% |
| **HPV vaccinations** | -- | 40.5% | **36.4%** |
| Mammography | 72.9% | 77.5% | 80.3% |
| Cervical screening | 80.0% | 83.9% | 79.5% |
| Colorectal screening | 89.4% | 93.4% | 91.7% |
| PSA testing | 31.8% | 33.3% | 33.4% |
| **Early-stage lung cancer** | -- | 30.2% | **26.7%** |
| **Note**: Shading indicates catchment area differs significantly from NYS or US. **Source**: ACS, BRFSS. | | | |
